# Acute ST Elevation Myocardial Infarction in Patients With Immune Thrombocytopenia Purpura: A Case Report

**DOI:** 10.4021/cr11w

**Published:** 2011-01-20

**Authors:** Sandeep K Dhillon, Edwin Lee, John Fox, Maurice Rachko

**Affiliations:** a Division of Cardiology, Department of Internal Medicine, University Hospital and Manhattan Campus for the Albert Einstein College of Medicine - Beth Israel Medical Center, New York, USA

**Keywords:** Acute myocardial infarction, Immune thrombocytopenic purpura, Percutanous coronary intervention, Platelet count, Asprin allergy

## Abstract

Acute myocardial infarction (AMI) in patients with immune thrombocytopenic purpura (ITP) is rare. We describe a case of AMI in patient with ITP. An 81-year-old woman presented with acute inferoposterior MI with low platelet count on admission (34,000/µl). Coronary angiography revealed significant mid right coronary artery (RCA) stenosis with thrombus, subsequently underwent successful percutaneous coronary intervention (PCI). In some patients with immune thrombocytopenia purpura and acute myocardial infarction, percutaneous coronary intervention is a therapeutic option.

## Introduction

Immune thrombocytopenia purpura (ITP) is an autoimmune disorder with a low platelet count and mucocutanous bleeding. The estimated incidence is 100 cases per 1 million persons per year [1]. The incidence of atherosclerosis and myocardial infarction in patients with congenital coagulation disorders and chronic thrombocytopenia is very low [2], and may be related to administration of intravenous immunoglobulin [3]. Due to the low incidence of acute myocardial infarctions in patients with immune thrombocytopenia purpura, there are few published data regarding the approach and management. We present a case of a patient with ITP and Asprin allergy who underwent percutaneous coronary intervention (PCI) and discuss the management of antiplatelet therapy.

## Case Report

An eighty-one-year-old woman without significant cardiovascular risk factors presented to the emergency department with chief compliant of acute onset of severe pressure-like, left-sided chest pain as she was walking back from church in the afternoon. It was associated with nausea but no vomiting, shortness of breath, diaphoresis, or palpitations. Prior to the onset of these symptoms she had been in her usual state of health and denied any cardiac complaints.

Her past medical history was significant for asthma, arthritis, and immune thrombocytopenic purpura (ITP) diagnosed one year ago by bone marrow biopsy with baseline platelet level 50,000 per µl requiring no treatment. She reported allergic reaction to aspirin which caused respiratory impairment and never underwent desensitization.

**Figure 1:**
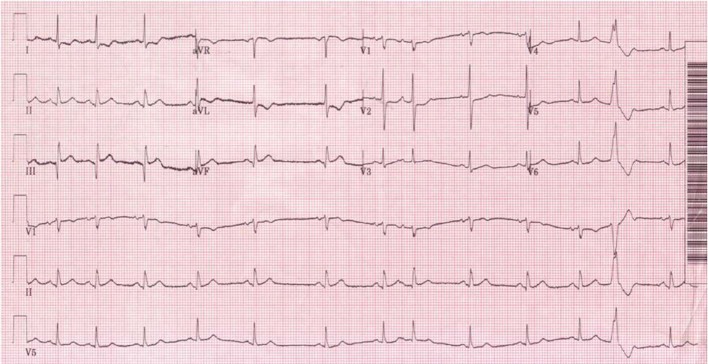
Initial presenting electrocardiogram: sinus rhythm with premature atrial and ventricular contractions, ST Elevation and Q waves in II, III, and aVF and tall R wave in V2 consistent with infero-post wall MI, STEMI.

**Figure 2:**
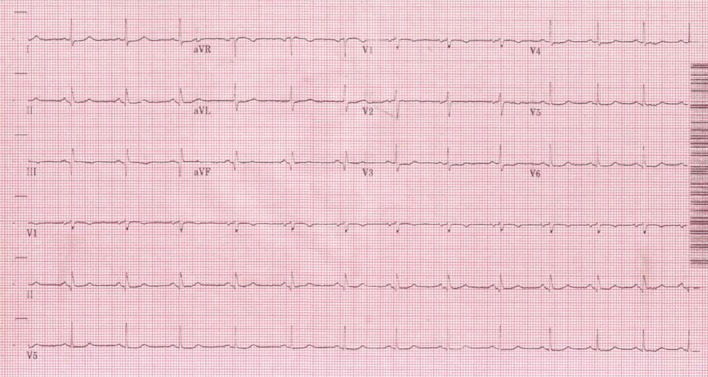
Follow-up electrocardiogram with formation of Q waves and normalization of the ST-T changes.

Upon arrival in the Emergency department chest pain had resolved. She was 5 feet 1 inch and 188 lbs; initial vital signs were blood pressure of 149/79 mmHg and heart rate of 88 bpm. The physical exam including cardiac exam was unremarkable. The electrocardiogram (ECG) (Fig. 1) revealed sinus rhythm with premature atrial and ventricular contractions, ST Elevation and Q waves in II, III, and aVF and tall R wave in V2 consistent with infero-post wall MI, STEMI. Laboratory results were significant for platelet count of 34,000 per µl, decreasing from baseline 50K (without any prior history of bleeding), and cardiac enzymes were as follows: Troponin I initial 0.3929 ng/ml and peaked at 10.9 ng/ml; Creatine Kinase (CPK) 666 U/L and peaked at 800 U/L with CK-MB and CK Index of 54.5 ng/ml and 7.9 respectively. The remainder of laboratory results, including coagulation profile, was normal at this time. The patient received Plavix 600 mg PO, Metoprolol 25 mg orally. An unfractionated heparin was used, with a loading dose of 5,100 international units (IU) intravenously then a continuous intravenous infusion of 13 U/hr with baseline PTT of 33.7 seconds, and transdermal nitroglycerin patch was placed during the emergency department course. Subsequent ECGs (Fig. 2) showed formation of Q waves and normalization of the ST-T changes, decision was made to medically treat the patient for the STEMI and plan for cardiac angiography since the patient was chest pain free.

**Figure 3:**
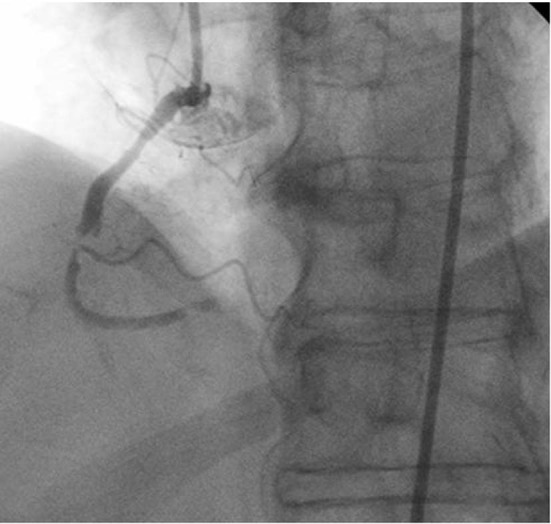
Right coronary artery (RCA) pre-intervention revealing 90% occlusion of the mid RCA with thrombus formation.

**Figure 4:**
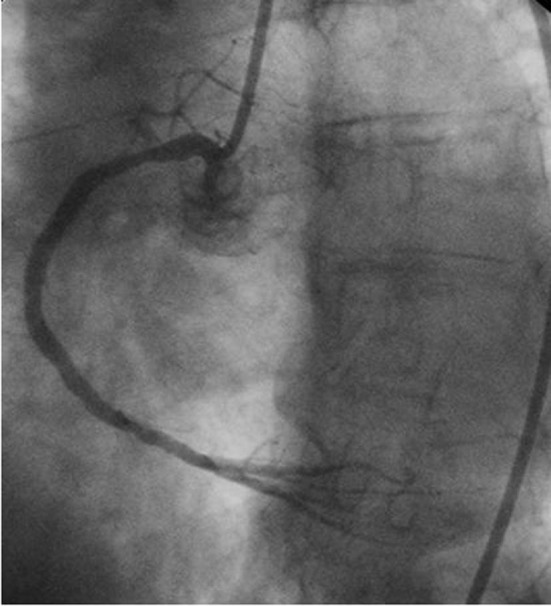
Right coronary artery (RCA) after bare metal implantation in the mid RCA.

The patient later underwent diagnostic coronary angiography via radial approach which revealed a 90% occlusion of the mid right coronary artery (RCA) with a TIMI 1 antegrade flow as well as thrombus formation (Fig. 3). It also revealed a 70% stenosis in the mid left circumflex (LCx) with an aneurysmal segment, a 75% stenosis in the proximal left anterior descending (LAD), a 70% stenosis in the diagonal (D2) ostium, and non-obstructive disease in the left main. The radial artery approach was uneventful, with the patient having a small proximal hematoma (1 cm x 2 cm approximately) that resolved with pressure dressing and warm compresses within the next 48 hours. Because the patient was stable and had history of aspirin allergy, she underwent aspirin desensitization while being on medical treatment pending staged percuteous coronary intervention (PCI) and stenting to the mid RCA lesion via femoral artery access. Later, the patient underwent successful desensitization of the aspirin and was started on a daily dose of 81 mg of aspirin. Repeat coronary angiography by femoral approach was performed for the PCI and bare metal stent was implanted in the mid RCA without any complications. The procedure was successfully concluded and final flow was TIMI 3 (Fig. 4). Her platelet level post-procedure upon discharge was 32,000 per µl and had ranged from 20K to 30K. The patient continued to tolerate aspirin 81 mg PO daily and Clopidogrel 75 mg PO daily three months post-procedure with stable platelet count.

## Discussion

ITP is primarily a disorder of increased platelet destruction mediated by autoantibodies to platelet-membrane antigen. It has been shown that despite the low platelet counts in patients with ITP, they can still form thrombosis. Atherosclerosis and acute myocardial infarction (AMI) are rare in patients with ITP, there are no precise recommendations for treatment. The pathogenesis of AMI in thrombocytopenic patients with ITP may stem from endothelial damage induced by autoantibodies presented on both platelet and coronary endothelial cells, since there is antigenic mimicry between platelets and endothelial cells [4].

PCI in patients with ITP requires special attention to the risks and benefits of platelet inhibition versus bleeding complications since one clinical problem of the disease is the bleeding tendency. Advanced age, history of bleeding, and refractory to treatment pose the highest risk of fatal bleeding. Medications that inhibit platelet function are generally not recommended. Though, after stent implantation, antiplatelet agents should be used in patients with CAD unless contraindication exists. This dilemma leads to difficulty in managing concomitant ITP and CAD.

Prior case reports of patients with chronic ITP have described successful management with aspirin, clopidogrel, and unfractionated heparin concurrently with no complications. They also described continuation of aspirin and clopidogrel upon discharge with no complications on follow-up and felt that aspirin, clopidogrel, and unfractionated heparin could be used with relative security [1]. One case attempted cutting balloon angioplasty, but eventually placed an intracoronary stent due to restenosis and treated with GP IIb/IIIa inhibitors. The patient was discharged on aspirin and clopidogrel in which the clopidogrel was discontinued due to a petechial rash [5]. Another case showed a patent stent by multislice computed tomography in a 71-year-old woman who had ITP at two year follow-up despite only taking aspirin 100 mg for 2 days and clopidogrel 75 mg daily for 7 weeks [2].

In each case, thoughtful clinical judgment was exercised, albeit with different approaches. It appears prudent to use a radial approach in patients with a positive Allen test. In all the reviewed cases that were intervened to choose to use a bare metal stent, in consideration of the length of time the patient would be on dual antiplatelet therapy. Also, using a vascular closure device would also appear to be wise as there was a case reporting a 72-year-old woman with ITP who suffered from a hematoma around the femoral puncture site that resulted in significant hypotension [6].

There is no obvious optimal approach, as some cases used GP IIa/IIIb while others deferred and some cases used intravenous immunoglobulin or steroids. Steroids and immunoglobulin therapy are indicated for ITP patients who have life threatening bleeding or will undergo an operation, particularly the elderly patients. However, intravenous immunoglobulin should be used with caution as it appears to be associated with increase in plasma viscosity which may induce myocardial infarction or thrombotic event [3]. Ultimately, treatment must be individualized by weighing the risks and benefits for each patient, but we have shown that aspirin, clopidogrel, and unfractionated heparin in patients with platelet counts in the range of 30,000/µl can be used safely. We even proceeded to desensitize our patient to Asprin in order to treat our patient with dual antiplatelet agents.

In conclusion, aspirin, clopidogrel, and unfractionated heparin can be used with relative security, but with strict control of coagulation times. When bare metal stent is chosen like our patient, to decrease the length of time the patient can take aspirin and clopidogrel. The current case suggests that primary PCI can be useful therapeutic strategy in acute STEMI with several precautions in managing the patient with ITP.
